# Andrographolide Stimulates Neurogenesis in the Adult Hippocampus

**DOI:** 10.1155/2015/935403

**Published:** 2015-12-22

**Authors:** Lorena Varela-Nallar, Sebastian B. Arredondo, Cheril Tapia-Rojas, Juan Hancke, Nibaldo C. Inestrosa

**Affiliations:** ^1^Centro de Investigaciones Biomédicas (CIB), Facultad de Ciencias Biológicas y Facultad de Medicina, Universidad Andrés Bello, Santiago, Chile; ^2^Centro de Envejecimiento y Regeneración (CARE), Departamento de Biología Celular y Molecular, Facultad de Ciencias Biológicas, Pontificia Universidad Católica de Chile, Santiago, Chile; ^3^Instituto de Farmacología y Morfofisiología, Universidad Austral de Chile, Valdivia, Chile; ^4^Center for Healthy Brain Ageing, School of Psychiatry, Faculty of Medicine, University of New South Wales, Sydney, Australia; ^5^Centro de Excelencia en Biomedicina de Magallanes (CEBIMA), Universidad de Magallanes, Punta Arenas, Chile

## Abstract

Andrographolide (ANDRO) is a labdane diterpenoid component of* Andrographis paniculata* widely used for its anti-inflammatory properties. We have recently determined that ANDRO is a competitive inhibitor of glycogen synthase kinase-3*β* (GSK-3*β*), a key enzyme of the Wnt/*β*-catenin signaling cascade. Since this signaling pathway regulates neurogenesis in the adult hippocampus, we evaluated whether ANDRO stimulates this process. Treatment with ANDRO increased neural progenitor cell proliferation and the number of immature neurons in the hippocampus of 2- and 10-month-old mice compared to age-matched control mice. Moreover, ANDRO stimulated neurogenesis increasing the number of newborn dentate granule neurons. Also, the effect of ANDRO was evaluated in the APPswe/PS1ΔE9 transgenic mouse model of Alzheimer's disease. In these mice, ANDRO increased cell proliferation and the density of immature neurons in the dentate gyrus. Concomitantly with the increase in neurogenesis, ANDRO induced the activation of the Wnt signaling pathway in the hippocampus of wild-type and APPswe/PS1ΔE9 mice determined by increased levels of *β*-catenin, the inactive form of GSK-3*β*, and NeuroD1, a Wnt target gene involved in neurogenesis. Our findings indicate that ANDRO stimulates neurogenesis in the adult hippocampus suggesting that this drug could be used as a therapy in diseases in which neurogenesis is affected.

## 1. Introduction

Andrographolide (ANDRO) is a labdane diterpenoid that is one of the main constituents of* Andrographis paniculata* [1, 2], a well-known medicinal plant widely used in Asia. ANDRO possesses a wide range of biological activities including anti-inflammatory action [3–5] and has also shown neuroprotective properties [6, 7]. More recently, it was also determined that ANDRO prevents neuropathological changes and improves spatial memory in the APPswe/PS1ΔE9 mouse model of Alzheimer's disease (AD) [8].

Regarding the mechanism of action of ANDRO, we recently determined that it directly inhibits the enzyme glycogen synthase kinase-3*β* (GSK-3*β*) [9], a key enzyme of the Wnt/*β*-catenin signaling pathway. The Wnt signaling pathway is activated by the binding of a Wnt ligand to seven-pass transmembrane receptors Frizzled, which may activate the canonical Wnt/*β*-catenin signaling pathway or noncanonical *β*-catenin-independent signaling cascades [10]. The Wnt signaling cascade regulates the development of the nervous system but also is an important modulator of adult nervous system regulating the formation of synaptic contacts, neurotransmission, and plasticity [11–13]. In addition, it has been more recently demonstrated that the Wnt signaling pathway regulates the formation of new neurons in the adult hippocampus (reviewed in [14]), a process known as adult neurogenesis.

Adult neurogenesis occurs mainly in two brain regions, the subventricular zone in the wall of the lateral ventricles and the subgranular zone (SGZ) in the hippocampal dentate gyrus [15, 16]. In the SGZ, neural progenitor cells located between the granule cell layer (GCL) and the hilus proliferate and give rise to neuroblasts that mature into granule cells that integrate into the hippocampal circuitry. These newborn neurons are relevant for hippocampal plasticity, learning, and memory [17–19]. Neurogenesis in the SGZ is controlled by the Wnt/*β*-catenin signaling pathway, which regulates proliferation, differentiation, and maturation of newborn neurons [20–23], effects mediated by the expression of Wnt target genes [24, 25]. Considering that ANDRO activates the Wnt signaling pathway and the transcription of Wnt target genes [9], in the present study we investigated the effect of ANDRO treatment on neurogenesis in the adult hippocampus. We determined that ANDRO increased cell proliferation and the generation of newborn mature granule cells in young and aged wild-type mice. In addition, ANDRO increased the proliferation and the density of immature neurons in APPswe/PS1ΔE9 mice. In both, wild-type and APPswe/PS1ΔE9 mice, ANDRO increased the activation of the Wnt signaling pathway in the hippocampus and increased the expression of the proneural Wnt target gene NeuroD1.

## 2. Materials and Methods

### 2.1. Animals and Treatment

Two- and ten-month-old wild-type C57BL/6 mice were injected intraperitoneally (i.p.) with 0.2% DMSO in saline solution (vehicle) alone or vehicle with 2 mg kg^−1^ ANDRO (Sigma-Aldrich) 3 times a week for 2, 4, or 6 weeks. Seven-month-old APPswe/PSEN1ΔE9 (stock #004462, The Jackson Laboratory) were injected i.p. with 2 mg kg^−1^ ANDRO or vehicle 3 times a week for 4 weeks. 5-Bromo-2′-deoxyuridine (BrdU, Sigma-Aldrich) was injected i.p. at 100 mg kg^−1^ for 1 or 3 days. All animals had access to water and food* ad libitum*, in a 12 : 12 h light/dark cycle. The Bioethical Committee of Pontificia Universidad Católica de Chile approved all procedures involving experimentation on animal subjects.

### 2.2. Perfusion and Postfixation

Animals were anesthetized (100 *μ*g ketamine + 10 *μ*g xylazine in 10 *μ*L saline/g) and then transcardially perfused with saline, followed by 4% paraformaldehyde (PFA, Sigma-Aldrich) in PBS. The brain was removed and placed in a vial with 4% PFA in PBS for 24 h at room temperature, dehydrated in 30% sucrose, and kept at 4°C until analysis.

### 2.3. Tissue Sectioning

After dehydration, brains were sectioned on a cryostat in 12 sets of serial coronal slices of 40 *μ*m thickness (Leica Microsystems) and collected in ice-cold PBS in multiwell dishes as previously described [26]. Each set contained 5–7 slices covering the entire length of the hippocampus and therefore corresponds to a representative sampling of the whole hippocampus.

### 2.4. Immunofluorescence

Immunodetection of BrdU and neuronal markers in tissue sections was carried out as previously described in [26]. Primary antibodies used were rat anti-BrdU (Abcam), rabbit anti-doublecortin (Cell Signaling Technology Inc.), monoclonal anti-NeuN (Millipore, Billerica, MA, USA), rabbit anti-Ki67 (Abcam), goat anti-NeuroD1 (Santa Cruz Biotechnology, Inc.), monoclonal anti-Nestin (Millipore), Alexa (Molecular Probes, Life Technologies), and DyLight (Abcam) conjugated secondary antibodies were used. Slices were mounted on gelatin-coated slides with Fluoromount-G (Electron Microscopy Sciences).

### 2.5. Image Analysis

For quantification, BrdU, Ki67, or DCX-positive cells were counted using a fluorescence microscope (Olympus BX51, Tokyo, Japan) as described in [26]. Briefly, total numbers of cells counted in all sections of 1 set of brain tissues (see tissue sectioning) were multiplied by the total number of sets to estimate the total number of BrdU, Ki67, or DCX-positive cells in the complete SGZ (BrdU and Ki67) or GCL (DCX). Double-labeled sections were analyzed by confocal laser microscopy (Olympus FV 1000). Image analysis and z-projections were made with ImageJ software (NIH, USA).

In APPswe/PSEN1ΔE9, the density of DCX-positive cells was estimated as previously described by Lie et al. [20] with some modifications. Briefly, DCX-positive cells in the GCL were counted in series of 5-6 random sections. NeuN immunoreactivity was used to measure the GCL volume. The area of the GCL was traced by using ImageJ software and a 10x objective. The density of immature neurons was expressed as DCX cells per volume (mm^3^) of dentate GCL.

### 2.6. Immunoblotting

Hippocampi were dissected on ice and either immediately frozen in liquid nitrogen or processed as previously described [27]. Briefly, hippocampi were homogenized in RIPA buffer (10 mM Tris/HCl pH 7.4, 5 mM EDTA, 1% NP-40, 1% sodium deoxycholate, and 1% SDS) supplemented with a protease inhibitor mixture (1 mM PMSF, 2 *μ*g/mL aprotinin, 1 *μ*g/mL pepstatin, and 10 *μ*g/mL benzamidine) and phosphatase inhibitors (25 mM NaF, 100 mM Na_3_VO_4_, 1 mM EDTA, and 30 *μ*M Na_4_P_2_O_7_), maintained on ice for 30 min before centrifugation at 20,000 g for 15 min at 4°C. Protein concentration in supernatants was determined using the BCA Protein Assay Kit (Pierce). Proteins were resolved in 10% SDS/PAGE, transferred to a PVDF membrane, reacted with primary antibodies overnight at 4°C, and then incubated with peroxidase-conjugated secondary antibodies (Pierce) and developed using the ECL technique (Western Lightning Plus ECL, PerkinElmer). Primary antibodies used were mouse anti-*β*-catenin, rabbit anti-GSK-3*β*, goat anti-NeuroD1, rabbit anti-*β*-tubulin (all from Santa Cruz Biotechnology, Inc.), and rabbit anti-GSK-3*β* pSer9 (Cell Signalling).

### 2.7. Statistical Analysis

Statistical analysis was performed using Prism 5 software (GraphPad Software Inc.). Statistical significance of differences was assessed using the unpaired Student's *t*-test; nonnormally distributed data was analyzed using the Mann-Whitney test. The number of animals per group in each experiment is indicated in the figure legends.

## 3. Results

### 3.1. ANDRO Stimulates Cell Proliferation in the SGZ of Adult Mice

**Figure 1:**
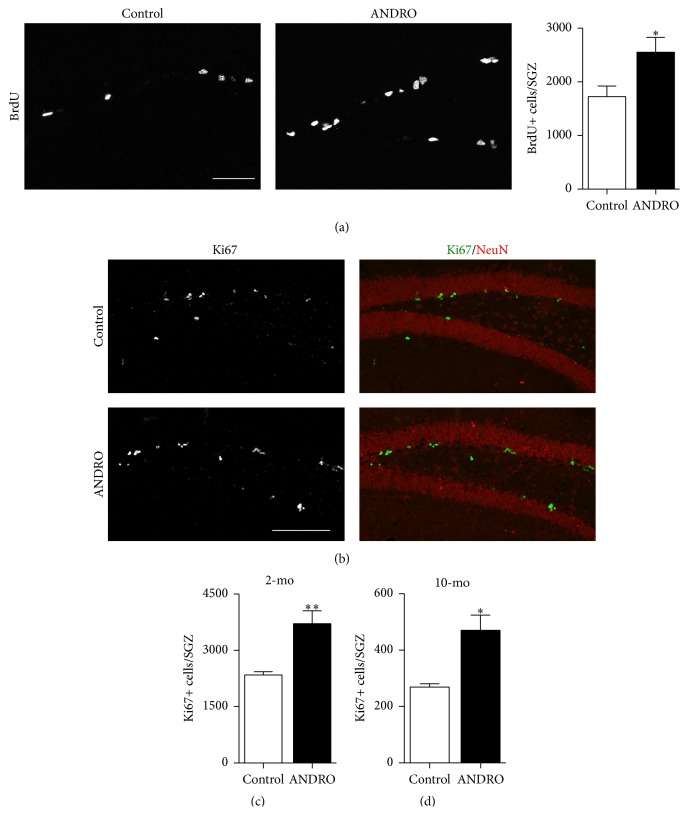
ANDRO induces proliferation in the dentate gyrus of adult mice. Two-month-old mice were injected i.p. with 2 mg kg^−1^ ANDRO or vehicle as control 3 times a week for 4 weeks, received a single dose of 100 mg kg^−1^ BrdU the last day of treatment, and were sacrificed 24 h after BrdU injection. (a) Images show representative immunostaining of BrdU. Scale bar: 50 *μ*m. The graph shows total number of BrdU-positive (BrdU+) cells in the whole SGZ of control and ANDRO-treated mice. Bars represent mean ± S.E. (*n* = 7 mice). (b) Representative immunostaining of Ki67 and NeuN. Scale bar: 100 *μ*m. (c, d) Total number of Ki67-positive (Ki67+) cells in the SGZ of control and ANDRO-treated 2-month-old (c) or 10-month-old (d) mice. Bars represent mean ± S.E. (*n* = 7 (c) and *n* = 5 (d) mice). ^*∗*^ *P* < 0.05, ^*∗∗*^ *P* < 0.01, Student's *t*-test.

To evaluate the effect of ANDRO on adult hippocampal neurogenesis, we first analyzed the effect of the drug on cell proliferation in the SGZ. Two-month-old mice were injected i.p. with 2 mg kg^−1^ ANDRO or vehicle as control, 3 times a week for 4 weeks. In the last day of the treatment, animals also received in the i.p. injection a single dose of the nucleotide analog BrdU (100 mg kg^−1^) and were sacrificed 24 h after the injection. The effect in cell proliferation was investigated by nuclear incorporation of BrdU (Figure 1(a)) and Ki67 staining (Figure 1(b)) used as a mitotic marker. BrdU immunoreactivity revealed that there was a significant increase in the total number of BrdU-positive cells in the SGZ of ANDRO-treated mice (Figure 1(a)) compared to control mice injected with the vehicle (control: 1,726 ± 196, ANDRO: 2,554 ± 275; *P* = 0.027). Also, there was a significant increase in the total number of Ki67-positive cells in the SGZ of mice treated with ANDRO compared to control mice (Figure 1(c), control: 2,341 ± 86, ANDRO: 3,711 ± 344; *P* = 0.001). These results indicate that ANDRO stimulates proliferation in the SGZ of adult mice.

Also, we evaluated the effect of ANDRO on cell proliferation in mice aged 10 months which show reduced levels of neurogenesis due to an age-related decline in neurogenesis observed in several species [28–31]. These mice were injected i.p. with 2 mg kg^−1^ ANDRO or vehicle as control, 3 times a week for 4 weeks; proliferation in the SGZ was evaluated by Ki67 staining. As expected, there was a strong decrease in Ki67-positive cells in 10-month-old mice compared with 2-month-old-mice (Figures 1(c) and 1(d)), and there was a significant increase in total number of Ki67-positive cells in mice treated with ANDRO (Figure 1(d), control: 269 ± 12, ANDRO: 470 ± 54; *P* = 0.011). Altogether, these results indicate that ANDRO increases cell proliferation in the dentate gyrus of young and aged mice.

### 3.2. ANDRO Increases Proliferation of Neural Progenitor Cells in the Adult Mouse Hippocampus

**Figure 2:**
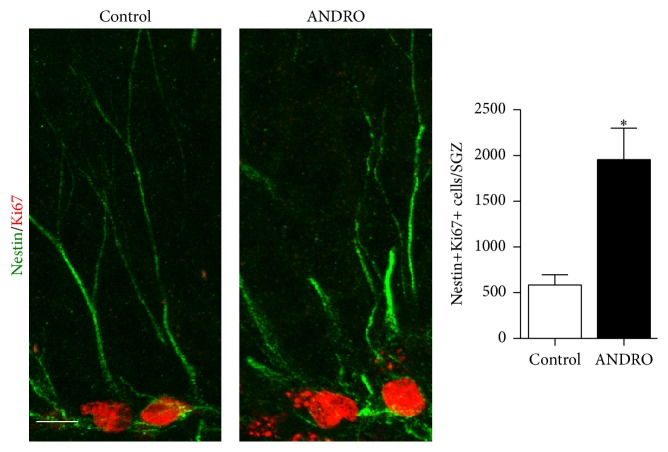
ANDRO induce proliferation of neural progenitors in the SGZ. Representative immunostaining of Ki67 and Nestin in 2-month old mice treated per 4 weeks with 2 mg kg^−1^ ANDRO or saline solution as control. Scale bar: 10 *μ*m. The graph represents quantification of the total number of Ki67-positive/Nestin-positive (Ki67+Nestin+) cells in the SGZ. Bars represent mean ± S.E. (*n* = 3 mice). ^*∗*^ *P* < 0.05, Student's *t*-test.

The increased number of BrdU- and Ki67-positive cells may result from increased proliferation of neural progenitor cells or neuroblasts. To evaluate whether quiescent neural progenitor cells are cellular targets of ANDRO, we evaluated Ki67 staining in Nestin-positive cells within the SGZ (Figure 2). Nestin is an intermediate filament expressed in neural progenitors but not in neuroblasts [32, 33]. Mice treated with 2 mg kg^−1^ ANDRO for 4 weeks showed a significant increase of Nestin+Ki67+ cells compared to vehicle-treated animals (Figure 2, control: 584 ± 112, ANDRO: 1,956 ± 343; *P* = 0.0191), indicating that the drug induced the activation of neural progenitors in the SGZ.

### 3.3. ANDRO Increases Neurogenesis in the Dentate Gyrus of Adult Mice

**Figure 3:**
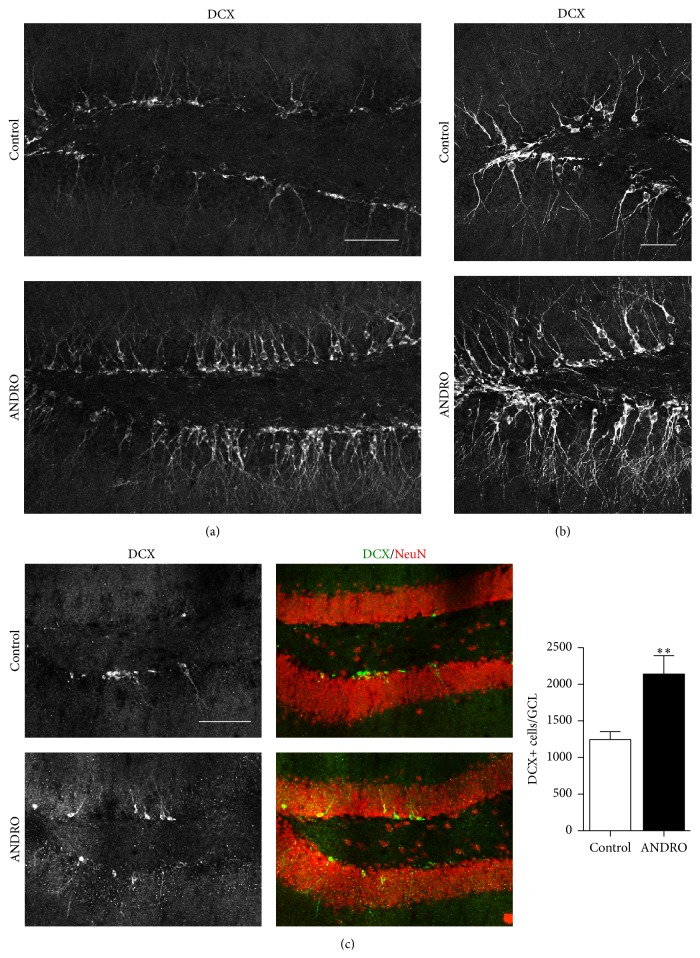
ANDRO increases the density of immature neurons in the dentate gyrus of adult mice. (a) Representative immunostaining of DCX in 2-month-old mice treated with vehicle or ANDRO for 4 weeks. Scale bar: 50 *μ*m. (b) Higher magnification of representative DCX immunostaining in control and ANDRO-treated mice. Scale bar: 30 *μ*m. (c) Representative images of double immunostaining for DCX and NeuN in 10-month-old mice treated with vehicle or ANDRO for 4 weeks. Scale bar: 100 *μ*m. Graph quantification of total number of DCX-positive (DCX+) cells in the GCL 10-month-old mice. Bars represent mean ± S.E. (*n* = 5 mice). ^*∗∗*^ *P* < 0.01, Student's *t*-test.

To evaluate whether ANDRO treatment induced the generation of new neurons in the adult dentate gyrus, first we carried out immunodetection of the immature neuronal marker doublecortin (DCX). A strong increase in the density of immature neurons positive for DCX was observed in the dentate gyrus of 2-month-old mice treated for 4 weeks with 2 mg kg^−1^ ANDRO compared with control mice injected with the vehicle (Figures 3(a) and 3(b)). Immunodetection of DCX was also carried out in 10-month-old mice that received the same treatment (Figure 3(c)). In agreement with the age-dependent decline in neurogenesis, there was an evident decrease in the density of immature DCX-positive neurons in mice aged 10 months compared with 2-month-old animals (compare Figures 3(a) and 3(c)). In 10-month-old mice, ANDRO treatment induced a significant increase in the total number of DCX-positive cells in the GCL compared with control mice injected with vehicle solution (Figure 3(c), control: 1,246 ± 108, ANDRO: 2,141 ± 250; *P* = 0.008).

**Figure 4:**
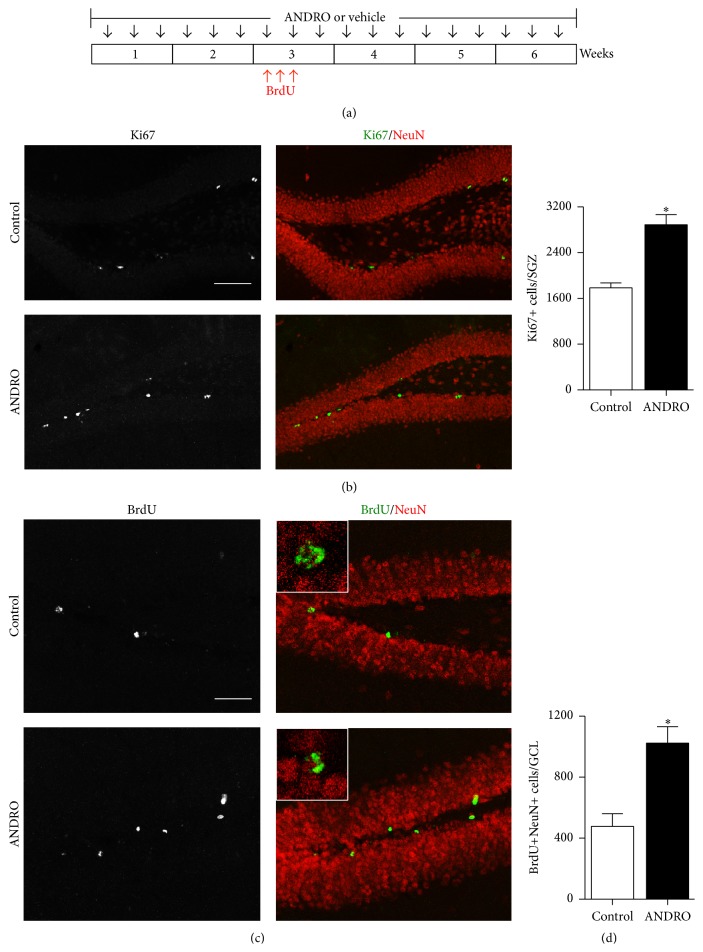
ANDRO increases the generation of newborn granule cells in the hippocampus of adult mice. (a) Schematic representation of the experimental procedure. Two-month-old mice were injected i.p. with 2 mg kg^−1^ ANDRO or vehicle as control 3 times a week for 2 weeks and then received a daily i.p. injection of 100 mg kg^−1^ BrdU for 3 consecutive days and continued with the treatments for 4 weeks. (b) Representative immunostaining of Ki67 and NeuN after 6 weeks of treatment. Scale bar: 100 *μ*m. The graph represents quantification of the total number of Ki67-positive (Ki67+) cells in the SGZ. (c) Representative immunostaining of BrdU and NeuN. Insets show higher magnifications of double-positive cells. Scale bar: 50 *μ*m (d) Quantification of the total number of double-positive (BrdU+NeuN+) cells in the GCL of control and ANDRO-treated mice. Bars represent mean ± S.E. (*n* = 4 mice). ^*∗*^ *P* < 0.05, Student's *t*-test.

To further evaluate whether ANDRO induced a net increase in neurogenesis, 2-month-old mice were injected i.p. with 2 mg kg^−1^ ANDRO or vehicle as control, 3 times a week for 2 weeks, and then received a daily i.p. injection of 100 mg kg^−1^ BrdU for 3 consecutive days and then continued with the injections of 2 mg kg^−1^ ANDRO or vehicle 3 times a week for 4 additional weeks (Figure 4(a)). As it was observed in the treatment of 4 weeks (Figure 1(c)), after 6 weeks of treatment there was a significant increase in the total number of Ki67-positive cells in the SGZ of mice treated with ANDRO compared with control mice (Figure 4(b); control: 1,784 ± 86, ANDRO: 2,888 ± 152; *P* = 0.0049), supporting the effect of the drug on cell proliferation. To assess the effect in neurogenesis, we evaluated the total number of newborn granule cells by analyzing the total number of BrdU-positive cells that were also positive for the mature neuronal marker NeuN. This was evaluated by confocal microscopy using z-plane sections to assess NeuN staining in each BrdU-positive cell (Figure 4(c), insets) and the total number of BrdU+NeuN+ cells was estimated in the whole dentate gyrus [26]. A significant increase was observed in the total number of BrdU+NeuN+ cells in the GCL of mice treated with ANDRO compared with control mice (Figure 4(d), control: 477 ± 84, ANDRO: 1,023 ± 108, *P* = 0.0286), indicating that ANDRO increased the generation of new granule neurons in the dentate gyrus. Altogether, these findings indicate that ANDRO increases neurogenesis in the adult hippocampus.

### 3.4. ANDRO Stimulates the Wnt/*β*-Catenin Signaling Pathway in the Hippocampus of Adult Mice

**Figure 5:**
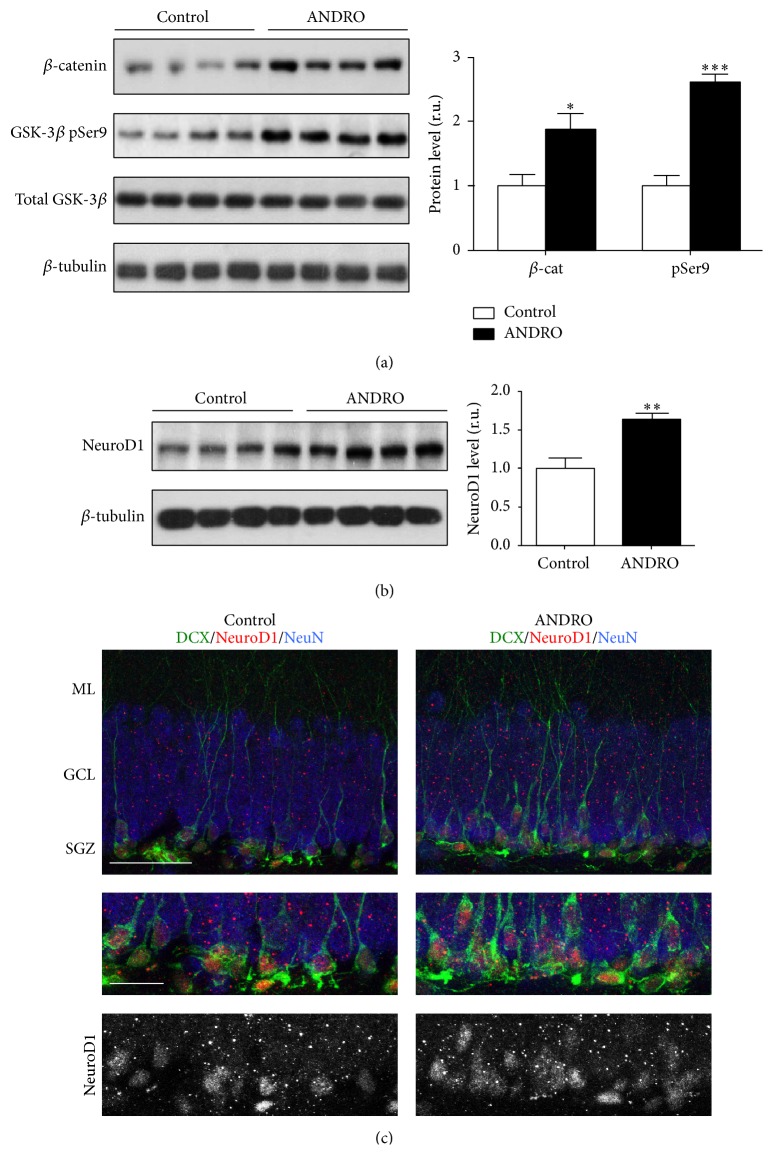
ANDRO induces the activation of the Wnt signaling pathway in the hippocampus of adult mice. (a) Immunoblots of *β*-catenin, inactive form of GSK-3*β* (phosphorylated in serine-9, pSer9), total GSK-3*β*, and *β*-tubulin used as loading control in total protein extracts obtained from the hippocampus of 2-month-old mice injected i.p. with 2 mg kg^−1^ ANDRO or vehicle as control 3 times a week for 4 weeks. The graph corresponds to the densitometric analysis of *β*-catenin and GSK-3*β* pSer9 normalized to *β*-tubulin and total GSK-3*β*, respectively. (b) Immunoblot of NeuroD1 and *β*-tubulin. The graph corresponds to the densitometric analysis of NeuroD1 normalized to *β*-tubulin level. (c) Representative immunostaining of NeuroD1, DCX, and NeuN in mice treated with vehicle or ANDRO for 4 weeks. Scale bar: 20 *μ*m. Bottom, higher magnifications of the images. Scale bar: 20 *μ*m. Bars represent mean ± S.E. (*n* = 4 mice). ^*∗*^ *P* < 0.05, ^*∗∗*^ *P* < 0.01, and ^*∗∗∗*^ *P* < 0.001, Student's *t*-test.

We previously showed that ANDRO inhibits the activity of GSK-3*β* [9], a component of the Wnt/*β*-catenin signaling pathway that upon activation of the pathway is inhibited, thereby preventing the phosphorylation of its target *β*-catenin which is translocated into the nucleus to activate the transcription of Wnt target genes [34]. We evaluated the activation of the Wnt signaling pathway in the hippocampus of 2-month-old mice injected i.p. with 2 mg kg^−1^ ANDRO or vehicle as control, 3 times a week for 4 weeks. As expected, we observed a significant increase in *β*-catenin level in the hippocampus of ANDRO-treated animals compared with control mice (Figure 5(a)), concomitantly with an increase in the level of the inactive form of GSK-3*β* phosphorylated in serine-9 residue (Figure 5(a)). In addition, we evaluated the levels of NeuroD1 which is a transcription factor involved in neurogenesis in the embryonic and adult brain [35] that was previously identified as a Wnt target gene [24]. A significant increase of NeuroD1 level was observed in the hippocampus of ANDRO-treated mice (Figure 5(b)). This increase was also observed by immunostaining (Figure 5(c)), in which NeuroD1 was mainly observed in DCX-positive cells, as previously described [35]. Altogether, these findings indicate that ANDRO induces the activation of the Wnt/*β*-catenin signaling pathway in the hippocampus of adult mice.

### 3.5. ANDRO Increases Neurogenesis in the Dentate Gyrus of APPswe/PSEN1ΔE9 Mice

**Figure 6:**
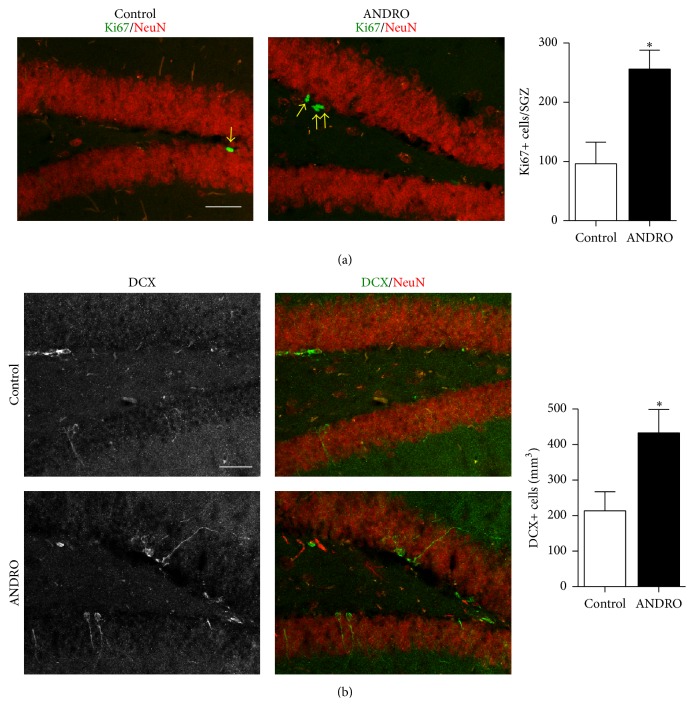
ANDRO stimulates proliferation and the density of immature neurons in the dentate gyrus of APPswe/PSEN1ΔE9. Seven-month-old APPswe/PSEN1ΔE9 mice were injected i.p. with 2 mg kg^−1^ ANDRO or vehicle as control 3 times a week per 4 weeks. (a) Representative images of double immunostaining for Ki67 and NeuN. The graph shows quantification of the total number of Ki67-positive (Ki67+) cells (yellow arrows) in the SGZ of mice treated with vehicle or ANDRO. Scale bar: 20 *μ*m. (b) Images show representative double immunostaining for DCX and NeuN. Scale bar: 20 *μ*m. Graph differences in the density of immature DCX-positive (DCX+) neurons in control and ANDRO-treated APPswe/PSEN1ΔE9 mice. Bars represent mean ± S.E. (*n* = 3 (a) or *n* = 5 (b) mice). ^*∗*^ *P* < 0.05, Student's *t*-test.

Finally, we evaluated the effect of ANDRO on neurogenesis in APPswe/PSEN1ΔE9 transgenic mouse model of AD that shows an impaired neurogenesis [26]. These double transgenic animals express the human amyloid precursor protein (APP) with the Swedish mutation (K595N/M596L) and presenilin 1 with the deletion of exon 9 and show histopathological hallmarks of AD (e.g., A*β* deposition, amyloid plaques, astrogliosis, and tau pathology) and cognitive impairment by 7 months of age [27]. We determined that at this age animals show a significant reduction in cell proliferation in the SGZ and show an impaired differentiation of newborn cells into DCX-positive neuroblasts [26]. APPswe/PSEN1ΔE9 mice at 7 months were injected i.p. with 2 mg kg^−1^ ANDRO or vehicle as control, 3 times a week for 4 weeks. Proliferation at the SGZ was evaluated by Ki67 staining (Figure 6(a)). Treatment with ANDRO strongly induced proliferation as determined by the increased number of Ki67-positive cells (APPswe/PSEN1ΔE9 control: 96 ± 37, APPswe/PSEN1ΔE9 ANDRO 256 ± 32; *P* = 0.05). In addition, an increased density of immature DCX-positive neurons was observed in mice treated with ANDRO compared to control transgenics that received vehicle injections (Figure 6(b)), indicating that the drug induced neurogenesis in APPswe/PSEN1ΔE9.

**Figure 7:**
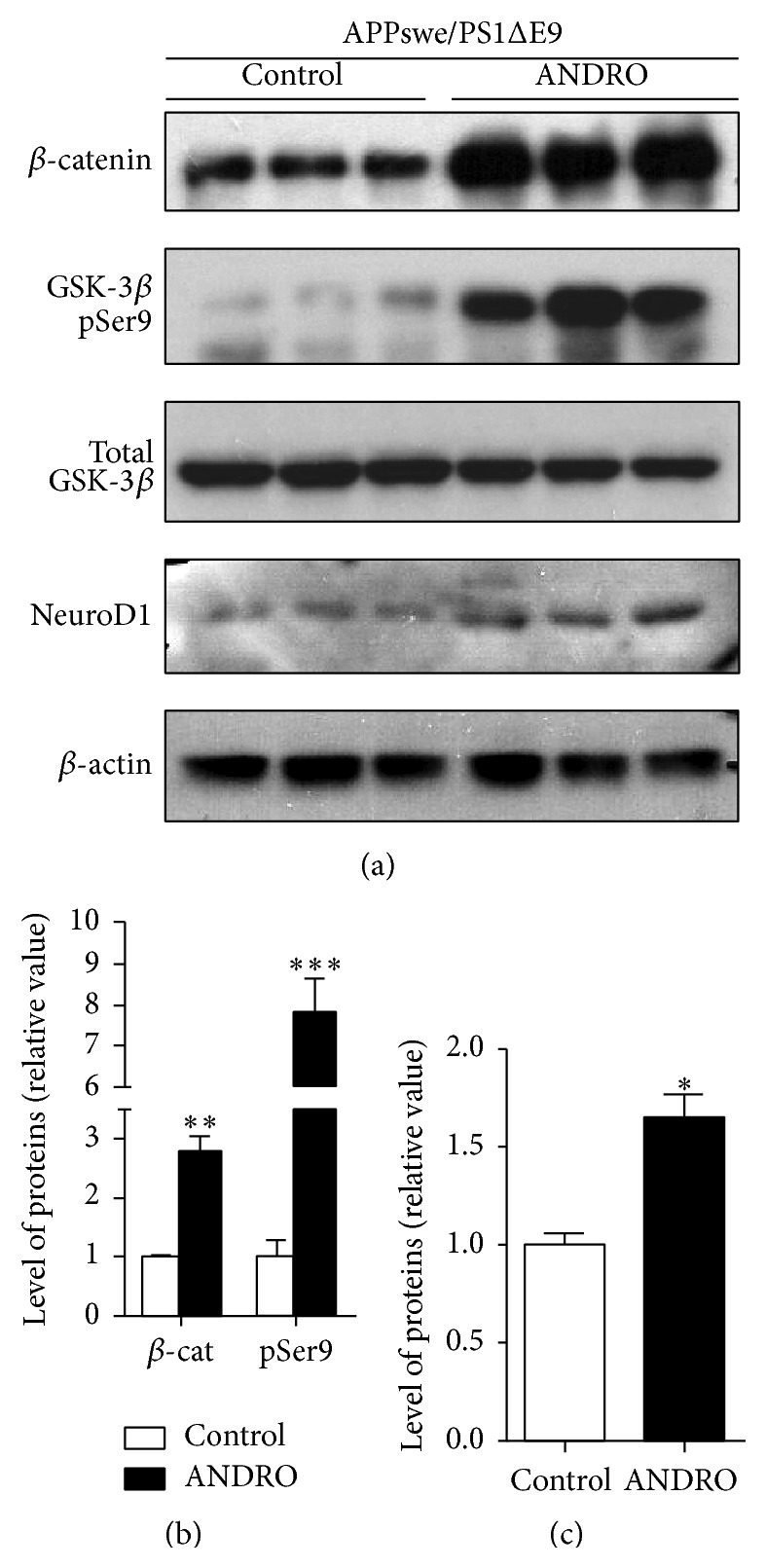
ANDRO increases NeuroD1 in the hippocampus of APPswe/PSEN1ΔE9. Seven-month-old APPswe/PSEN1ΔE9 mice were injected i.p. with 2 mg kg^−1^ ANDRO or vehicle as control 3 times a week per 4 weeks. (a) Immunoblots of *β*-catenin, inactive form of GSK-3*β* (phosphorylated in serine-9, pSer9), total GSK-3*β*, NeuroD1, and *β*-actin in total protein extracts obtained from the hippocampus of control and ANDRO-treated APPswe/PSEN1ΔE9 mice. (b) Densitometric analysis of *β*-catenin and GSK-3*β* pSer9 normalized to *β*-tubulin and total GSK-3*β*, respectively. (c) Densitometric analysis of NeuroD1 normalized to *β*-tubulin. Bars represent mean ± S.E. (*n* = 3 mice). ^*∗*^ *P* < 0.05, ^*∗∗*^ *P* < 0.01, and ^*∗∗∗*^ *P* < 0.001, Student's *t*-test.

In these mice, we also evaluated the Wnt target gene NeuroD1 (Figure 7(a)). As we previously observed in 12-month-old transgenic mice [8], increased levels of *β*-catenin and GSK-3*β* phosphorylated in serine-9 were observed in APPswe/PSEN1ΔE9 mice treated with ANDRO compared with control mice (Figure 7(b)). In addition, and as observed in wild-type animals (Figure 5(b)), ANDRO treatment induced a significant increase of NeuroD1 level at the hippocampus of APPswe/PSEN1ΔE9 mice (Figure 7(c)).

## 4. Discussion

The generation of new neurons in the hippocampus during adulthood has been determined in several species and has shown to contribute to the plasticity of the hippocampus and to some hippocampal processes including spatial learning and memory [17, 19]. This process is compromised in several pathologies affecting the central nervous system (e.g., AD, schizophrenia, and mood disorders) and also is affected during normal aging [30, 36]; the decreased neurogenesis has been linked, for example, to cognitive deficits associated with some of these conditions; therefore, it is tempting to search for new approaches to stimulate this process in normal and diseased brain. Here, we evaluated the ability of ANDRO, one of the active components of the medicinal plant* Andrographis paniculata*, to stimulate neurogenesis, and demonstrated that ANDRO induces proliferation and the generation of new neurons in the dentate gyrus of the adult hippocampus in wild-type mice and in a mouse model of AD.

In the adult dentate gyrus, new granule cells are continuously being generated from neural progenitor cells that are located between the hilus and the GCL. After activation, these cells give rise to transit-amplifying progenitors or intermediate progenitor cells that then commit to the neuronal fate generating neuroblasts that develop into immature neurons that extend dendrites to the GCL and molecular layer and project their axons to the CA3 region [16]. These newborn neurons will mature during several weeks into functional dentate granule neurons that form synaptic connections and become integrated into the hippocampal circuitry [37, 38]. We determined that ANDRO treatment for 4 weeks stimulated proliferation in the SGZ and increased the density of immature neurons in the GCL. Our results showed that ANDRO increased the activation of quiescent neural progenitor cells, strongly suggesting that these are the cellular target of ANDRO activity. Interestingly, the effects of ANDRO on proliferation and immature neurons were observed in mice aged 2 months at the beginning of the treatment and also in mice at 10 months of age where neurogenesis is significantly reduced. An age-dependent decline in hippocampal neurogenesis has been evidenced in different species including humans [28–31]; however, studies reveal that neurogenesis can be stimulated even at advanced stages of aging. The effect of ANDRO in 10-month-old mice indicates that the drug is able to stimulate neurogenesis in aged mice. Moreover, we determined that ANDRO induced a net increase in neurogenesis. The expression of mature neuronal markers by newborn neurons takes about four weeks [16]; we determined that ANDRO increased the total number of newborn cells expressing the mature neuronal marker NeuN (evaluated four weeks after administration of BrdU), indicating that the drug induced a net increase in the total number of newly born granule neurons. Altogether, these findings indicate that ANDRO stimulates neurogenesis in the adult hippocampus.

The effect of ANDRO on neurogenesis may involve the inhibition of GSK-3*β*. We recently determined that ANDRO inhibits GSK-3*β* by a substrate-competitive mode of action [9]. Interestingly, ANDRO shows high selectivity for GSK-3*β*, since it had no effect on cyclin-dependent kinase 5 (Cdk5), Ca^2+^/calmodulin-dependent protein kinase II (CaMKII), extracellular signal-regulated kinase (ERK), c-Jun N-terminal kinase (JNK), protein kinase C (PKC), Akt, casein kinase (CK), and S6 kinase [9]. Stimulation of neurogenesis by GSK-3*β* inhibition has been reported* in vitro* and* in vivo*. Treatment of cultured adult hippocampal progenitors with the GSK-3*β* inhibitor lithium induced proliferation [39] and* in vivo* treatment with lithium induced proliferation and neuronal fate specification in the hippocampus of a mouse model of AD [40]. In addition, an impaired neurogenesis was observed in a GSK-3 knock-in mouse carrying mutations to block inhibitory phosphorylation of the kinase [41] and in mice overexpressing GSK-3*β* [42] which also show morphological alterations in newborn neurons [43]. Therefore, it is likely that ANDRO may stimulate neurogenesis via inhibition of GSK-3*β*. Other mechanisms that have been associated with the biological effects of ANDRO include inhibition of PI3K/Akt and NF-*κβ* pathways [44–46]. As mentioned, we previously determined that ANDRO has no effect on Akt activity [9], and since inhibition of NF-*κβ* signaling pathway is associated with an impaired adult hippocampal neurogenesis [47, 48], it is unlikely that the positive effect of ANDRO on neurogenesis may involve these cascades.

As previously mentioned, GSK-3*β* is a key component of the Wnt/*β*-catenin signaling pathway, which has been shown in several* in vivo* studies to regulate proliferation, differentiation, and maturation of adult-born granule neurons [20–22, 24]. Through the inhibition of GSK-3*β*, ANDRO is a potent activator of the Wnt signaling pathway [9]. Here, we determined that concomitantly with the increase in neurogenesis ANDRO treatment induced the Wnt/*β*-catenin signaling pathway in the hippocampus of adult mice, as determined by the increase in *β*-catenin protein and the increase in the level of the inactive form of GSK-3*β*. In addition, we observed increased level of NeuroD1, a previously described Wnt target gene [24] that is needed for the survival and maturation of adult-born neurons. Therefore, it might be suggested that the effects of ANDRO may be mediated by the activation of the Wnt signaling pathway and the expression of proneural Wnt target genes such as NeuroD1. Interestingly, increased level of NeuroD1 was also observed in ANDRO-treated APPswe/PS1ΔE9 mice. In this mouse model of AD, which shows reduced levels of proliferation in the SGZ and decreased differentiation of neural progenitor cells into neurons compared with age-matched wild-type mice [26, 49], we determined that ANDRO treatment induced almost 3-fold increase in cell proliferation in the SGZ. We had observed such a strong effect in cell proliferation in this transgenic mouse by exposure to voluntary wheel running [50], which is a well-known potent inductor of neurogenesis in young and aged mice [51, 52]. Also, we determined that ANDRO treatment increased the density of immature neurons in APPswe/PS1ΔE9 mice, indicating that the drug induced neurogenesis.

## 5. Conclusions

In the present study, we have demonstrated that ANDRO, the active component of the medicinal plant* Andrographis paniculata*, stimulates adult hippocampal neurogenesis. Previously, ANDRO showed neuroprotective effects in a rat model of permanent cerebral ischaemia [6] and against oxidative damage induced by nicotine [7]; in addition, in the APPswe/PS1ΔE9 mouse model of AD, ANDRO prevented neuropathological changes associated with the disease and improved spatial memory [8]. Here, we showed that ANDRO induces proliferation and the generation of new neurons in the adult hippocampus of wild-type and APPswe/PS1ΔE9 mice, providing new evidence to suggest ANDRO as a potential therapeutic drug for the treatment of brain diseases.
